# Assessing the quality of care in sick child services at health facilities in Ethiopia

**DOI:** 10.1186/s12913-020-05444-7

**Published:** 2020-06-23

**Authors:** Theodros Getachew, Solomon Mekonnen Abebe, Mezgebu Yitayal, Lars Åke Persson, Della Berhanu

**Affiliations:** 1 Health System and Reproductive Health Research Directorate, Ethiopian Public Health Institute, Addis Ababa, Ethiopia; 2 Institute of Public Health, College of Medicine and Health Science, University of Gondar, Gondar, Ethiopia; 3 London School of Hygiene and Tropical Medicine, London, UK

**Keywords:** Quality, Satisfaction, Child health, Integrated management of childhood illness, Ethiopia

## Abstract

**Background:**

Quality of care depends on system, facility, provider, and client-level factors. We aimed at examining structural and process quality of services for sick children and its association with client satisfaction at health facilities in Ethiopia.

**Methods:**

Data from the Ethiopia Service Provision Assessment Plus (SPA+) survey 2014 were used. Measures of quality were assessed based on the Donabedian framework: structure, process, and outcome. A total of 1908 mothers or caretakers were interviewed and their child consultations were observed. Principal component analysis was used to construct quality of care indices including a structural composite score, a process composite score, and a client satisfaction score. Multilevel mixed linear regression was used to analyze the association between structural and process factors with client satisfaction.

**Result:**

Among children diagnosed with suspected pneumonia, respiratory rate was counted in 56% and temperature was checked in 77% of the cases. A majority of children (92%) diagnosed with fever had their temperature taken. Only 3% of children with fever were either referred or admitted, and 60% received antibiotics. Among children diagnosed with malaria, 51% were assessed for all three Integrated Management of Childhood Illnesses (IMCI) main symptoms, and 4% were assessed for all three general danger signs. Providers assessed dehydration in 54% of children with diarrhea with dehydration, 17% of these children were admitted or referred to another facility, and Oral Rehydration Solution was prescribed for 67% while none received intravenous fluids. The number of basic amenities in the facility was negatively associated with the clients’ satisfaction. Private facilities, when the providers had got training for care of sick children in the past 2 years, had higher client satisfaction. There was no statistical association between structure, process composite indicators and client satisfaction.

**Conclusion:**

The assessment of sick children was of low quality, with many missing procedures when comparing with IMCI guidelines. In spite of this, most clients were satisfied with the services they received. Structural and process composite indicators were not associated with client’s satisfaction. These findings highlight the need to assess other dimensions of quality of care besides structure and process that may influence client satisfaction.

## Background

In spite of global progress in child survival, 5.4 million children died before the age of 5 years in 2017, whereof most deaths occurred in low- and middle-income countries [1]. Ethiopia was one of few low-income countries achieving the fourth Millennium Development Goal by a two-thirds reduction of under-five mortality from 1990 to 2015 [2]. Nonetheless, Ethiopia remains one of the Sub-Saharan countries with the highest burden of child deaths [3].

Universal health coverage has been recommended as a strategy to improve health of a population. The success of this strategy is, however, also dependent on the provision of good-quality health care [4]. Poor quality of care provided at health facilities may contribute to child mortality [4, 5]. Poor-quality care can also lead to other adverse outcomes, including lack of trust and confidence in the health system [6].

In an effort to improve the quality of child health services, the World Health Organization (WHO) and the United Nations International Children’s Emergency Fund (UNICEF) developed the Integrated Community Case Management (iCCM) of childhood illnesses strategy [7]. This strategy aims to reduce morbidity and mortality among children under the age of 5 years through improved health workers’ skills by training and supportive supervision; improved health systems, including equipment, supplies, organization of work and referral systems; and improved key family practices and child care at community and household levels [8].

In Ethiopia, health centers and some hospitals provide Integrated Management of Newborn and Childhood Illness (IMNCI) and at health post level these services are referred to as the Integrated Community Case Management (iCCM). The Health Extension Workers (HEW) at health posts manage pneumonia, diarrhea, malaria, and malnutrition and refer severe cases [9].

As many countries attempt to improve service delivery, there is an increased need to assess the quality of care at health facilities, in order to identify problems and to identify factors that could lead to better care. The concept of quality of health care, in view of its subjective nature, is difficult to define and consequently difficult to measure. Donabedian proposed the triad of structure, process, and outcome to evaluate the quality of health care [10]. Based on Donabedian’s framework, this study examined the quality of sick child services and determinants of client satisfaction at health facilities in Ethiopia. Few studies have assessed the quality of care provided in Ethiopia [11, 12]. However, none of these studies examined the association between structural and process quality dimensions and client satisfaction. Thus, this study aimed at evaluating the quality of care for sick children at Ethiopian health facilities by assessing all three components of the Donabedian framework: structure, process, and outcome.

## Methods

### Data source

Data used in this study came from the Ethiopian Service Provision Assessment Plus survey (SPA+). Data were collected from March to July, 2014. The survey included observations of services provided to sick children to assess to what extent providers adhered to accepted service delivery and quality standards. Exit interviews were conducted with mothers or caretakers of sick children, whose consultations had been observed. The exit interviews included questions on the clients’ perception of the service delivery environment. Detailed methodology on SPA+ has previously been published [13].

In this survey, all hospitals and a representative sample of health centers, clinics, and health posts were selected. In each facility, clients were systematically selected for observation based on the number of clients visiting the facility for each of the sick child services on the day of the survey. A maximum of five clients were observed for a maximum of three providers of sick child services, with a maximum of 15 observations in a given facility. Interviewers were attempting to conduct an exit interview with all caretakers of observed sick children.

### Measurements

Based on Donabedian’s quality of care framework, this study assessed three aspects of the quality of care: structure, process, and outcome [14]. In addition, descriptive statistics on adherence to IMCI guidelines for the assessment, physical examination, and treatment of sick children were presented. Structural data came from facility inventory interviews, while process measurements primarily depended on direct observations of client-provider interactions. Information about the clients’ satisfaction, which is viewed as reflecting outcome, was based on exit interviews with mothers or caregivers.

#### Information about structure

Table 1 shows structure and process indicators, which were selected based on the WHO Service Availability and Readiness Assessment (SARA) reference manual [15]. The structural indicators of quality of care included the facilities’ management system, service availability, infrastructure, and equipment.

**Table 1:** Items used in defining the structure and process indicators for quality in sick child services in Ethiopia. Ethiopian Service Provision Assessment Plus Survey 2014

	Definition	Variable type	
		Categorical	Continuous
**STRUCTURE**			
Routine management meetings	Whether there are monthly meeting to discuss management issues	Yes/No	–
System to collect client opinion	Whether the facility has a system to obtain clients’ opinions regarding services	Yes/No	–
Quality assurance system	Whether the facility has a routine quality assurance system	Yes/No	–
Supervision	Whether the facility reported that the last supervision visit was in the last 6 months	Yes/No	–
Basic amenities	Number of amenities at facility: water, electricity, generator, telephone, internet, ambulance	–	0–6
Health workers always available	24 h staff availability	Yes/No	–
Infection prevention precautions	Number of infection prevention measures at facility: sharps containers, gloves, disinfectant, disposable needles or autodestruct syringes with disposable syringes, waste bin, hand disinfectant	–	0–5
Equipment available	Number of pieces of sick child equipment available at facility: Infant scale, child scale, thermometer, stethoscope, timer/watch or clock	–	0–5
IMCI guide followed	Whether the facility always follows guide for integrated management of childhood illness (IMCI) when assessing/treating sick child	Yes/No	
Malaria diagnosis and treatment	Whether the facility always has malaria blood tests available for children under age 5	Yes/No	
IMCI mother’s card	Whether IMCI mother’s card are available at facility	Yes/No	
**PROCESS**			
Number of symptoms checked	Number of symptoms that the provider asked for or that the caregiver mentioned: Cough or difficult breathing, diarrhea, fever or body hotness, ear problems, unable to drink or breastfeed, vomiting everything, convulsions	–	0–7
Physical examination of sick child	Number of types of sick child exams performed: Took child’s temperature by thermometer, Felt the child for fever or body hotness, Counted respiration (breaths) for 60 s, Auscultated child (listen to chest with stethoscope) or count pulse, Checked skin turgor for dehydration (e.g., pinch abdominal skin), Checked for pallor by looking at palms, Checked for pallor by looking at conjunctiva, Looked into child’s mouth, Checked for neck stiffness, Looked in child’s ear, Felt behind child’s ear, Undressed child to examine (up to shoulders/down to ankles), Pressed both feet to check for edema, Weighed the child, Plotted weight on growth chart, Checked for enlarged lymph nodes in 2 or more of the following sites: neck, axillae, groin	–	0–16
Information provided to caregiver	Number of pieces of information given to caregiver during consultation: provide general information about feeding/breastfeeding, advise extra fluids during this sickness, advise continued feeding during sickness, name the illness for the caretaker, describe symptoms requiring immediate return for care	–	0–5
Provider used visual aids	Whether provided used visual aids during consultation	Yes/No	–
Provider discussed follow-up visit	Whether the provider discussed follow-up visit during consultation	Yes/No	–

#### Information about process

The process indicators of quality of care consisted of both interpersonal and technical features of the provider-client interactions. The interpersonal characteristics include information provided to a caregiver and the technical characteristics of the provider-client interactions included symptoms checked and physical examination conducted. These were based on the IMCI guidelines [16] and were derived from direct observation of the care provided during the consultations with sick children (Table 1).

#### Information about client satisfaction

Client satisfaction with sick child services, representing the outcome, was measured in the exit interview using 11 questions about mothers’ or caretakers’ perceptions of the quality of care. Satisfaction was rated as an index of problems *not* encountered during the visit.

### Statistical analyses

Principal component analysis was used to construct several indices including the structural composite score, the process composite score, and the client satisfaction score. In doing so, we generated the indices with the highest Cronbach’s alpha, keeping the maximum number of common variables. The scores were computed based on the first principle component, which explained the largest proportion of the total variance. The structure, process, and outcome quality of care indicators and their respective composite indices were computed. The responses of caregivers were aggregated into an index to measure satisfaction using principal components analysis. Cronbach’s alpha of 0.82 revealed that items co-varied and probably measured the same underlying concept, i.e., satisfaction. In this study, a high reliability implies that it measures client satisfaction, while low reliability indicates that it measures something else (or possibly nothing at all).

Multilevel mixed linear regression was used to analyze if structural and process indices were associated with client satisfaction. The fixed effects, which is a measure of association, and random effects that is a measure of variation for client satisfaction, were determined by considering the region as the level of variation in the multilevel mixed regression. Four models were fitted, Model I included structural indicators, Model II included process indicators, Model III included each of the structure and process items, and Model IV included structural and process composite indicator. In the regression analysis for client satisfaction, we controlled for provider level covariates that could affect client satisfaction. The Akaike’s information criterion (AIC) and Bayesian information criterion (BIC) were used to measure the model fit and complexity. The model with the smallest value of the information criterion was considered to be better. The STATA Statistical Software version 14.2 (Stata Corp LP, College Station, TX, USA) was used to conduct these analyses.

## Results

**Fig. 1:**
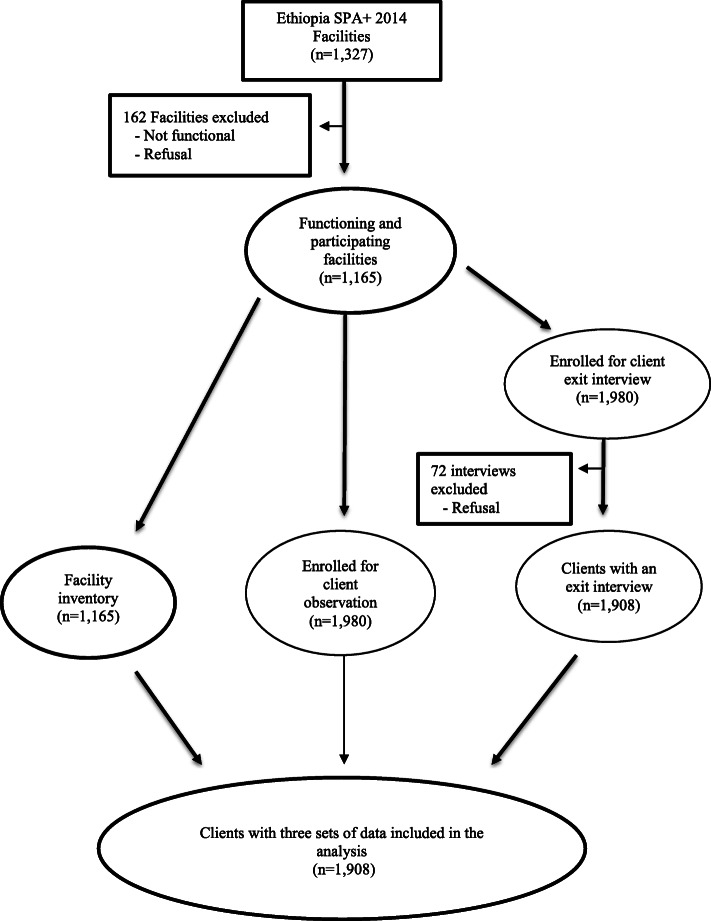
Study flow for the analysis of determinants of client satisfaction in sick child services in Ethiopia. Ethiopian Service Provision Assessment Plus Survey 2014

A total of 1908 clients were observed and interviewed at 1165 health facilities (hospitals, health centers, clinics, and health posts). Facility data were linked to data regarding sick children, whose consultations were observed and their mothers or caregivers, who were interviewed (Fig. 1).

### Client characteristics

The clients’ characteristics are shown in Table 2. The majority of the clients’ observations and exit interviews were performed in Oromia region (21%) followed by Amhara region (15%). The majority was done in urban areas (72%), primarily in hospitals (46%) and health centers (38%).

**Table 2:** Distribution of observed sick child consultations and exit interviews with mothers or caretakers by background characteristics. Ethiopian Service Provision Assessment Plus Survey 2014

Facility characteristics	Total number of clients interviewed	Percent
**Region**		
Addis Ababa	255	13.4
Afar	70	3.7
Amhara	284	14.9
Benishangul Gumuz	115	6.0
Dire Dawa	121	6.3
Gambella	38	2.0
Harari	100	5.2
Oromia	403	21.1
SNNP	229	12.0
Somali	105	5.5
Tigray	188	9.9
**Facility type**		
Hospitals	876	45.9
Health centers	720	37.7
Health posts	65	3.4
Clinic	247	12.9
**Urban/rural**		
Urban	1382	72.4
Rural	526	27.5
**Total**	1908	100

### Description of sick child service quality attributes

Table 3 presents the description and comparisons of sick child service quality attributes across the different types of facilities. About half (53%) of the providers in the facilities had received sick child care training in the past 2 years. Among the structural indicators, 91% had a system to conduct routine management meetings, 82% had a system to collect client opinions, but only 33% used the IMCI mother’s card. The purpose of that card was to provide the mother with reminders of the key messages she had received from the health worker. Only 2% of the sick child care providers had used visual aids during the consultations. More than two thirds (67%) of the clients reported that waiting time to see the provider was not a problem, and 74% of the clients reported that cost of the services was not a problem.

**Table 3:** Sick child services quality attributes by facility type. Ethiopian Service Provision Assessment Plus Survey 2014

FACILITY / PROVIDER CHARACTERISTICS	Hospitals		Health centers		Health posts		Clinics		Total	
	N	%	N	%	N	%	N	%	N	%
Facility managing authority										
Public	697	79.6	715	99.3	65	100.0	1	.4	1478	77.5
Private/Non-Governmental Organization	179	20.4	5	0.7	0	0.0	246	99.6	430	22.5
Urban/Rural										
Urban	781	89.2	396	55.0	6	9.2	199	80.6	1382	72.4
Rural	95	10.8	324	45.0	59	90.8	48	19.4	526	27.6
Provider in the facility received sick child care training in past 2 years	442	50.5	467	64.9	40	61.5	52	21.1	1001	52.5
STRUCTURE COMPOSITE INDEX										
Routine management meetings	866	98.9	704	97.8	43	66.2	118	47.8	1731	90.7
System to collect client opinion	823	93.9	570	79.2	18	27.7	145	58.7	1556	81.6
Quality assurance system	726	82.9	485	67.4	22	33.8	57	23.1	1290	67.6
Supervision	813	92.8	693	96.3	55	84.6	228	92.3	1789	93.8
Health workers always available	868	99.1	702	97.5	26	40.0	108	43.7	1704	89.3
IMCI guide followed	608	69.4	653	90.7	43	66.2	96	38.9	1400	73.4
Diagnose and/or treat malaria	866	98.9	717	99.6	63	96.9	238	96.4	1884	98.7
Number of basic amenities (mean, SE)	5 (0.028)		3 (0.047)		2 (0.093)		3 (0.076)		4 (0.033)	
Number of infection prevention precautions (mean, SE)	3 (0.040)		3 (0.050)		4 (0.144)		4 (0.073)		3 (0.03)	
Number of sick child equipment available (mean, SE)	4 (0.037)		3 (0.039)		3 (0.133)		3 (0.065)		4 (0.025)	
IMCI mother’s card	329	37.6	263	36.5	17	26.2	11	4.5	620	32.5
Cronbach’s alpha									0.4613	
PROCESS COMPOSITE INDEX										
Provider used visual aids	10	1.1	28	3.9	0	0.0	1	.4	39	2.0
Provider discussed follow-up visit	133	15.2	183	25.4	19	29.2	61	24.7	396	20.8
Number of symptoms checked (mean, SE)	3 (0.051)		4 (0.058)		3 (0.204)		4 (0.092)		3 (0.035)	
Number of physical examinations of sick child (mean, SE)	4 (0.076)		3 (0.076)		3 (0.229)		4 (0.149)		4 (0.05)	
Number of information provided to caregiver (mean (SE)	1 (0.044)		1 (0.054)		1 (0.114)		1 (0.085)		1 (0.031)	
Cronbach’s alpha									0.4588	
OUTCOME / SATISFACTION										
Time you waited to see provider	514	58.7	522	72.5	49	75.4	195	78.9	1280	67.1
Ability to discuss problems or concerns about your health with the provider	590	67.4	517	71.8	51	78.5	184	74.5	1342	70.3
Amount of explanation you received about the problem or treatment	593	67.7	501	69.6	50	76.9	181	73.3	1325	69.4
Privacy from having others see the examination	763	87.1	611	84.9	53	81.5	219	88.7	1646	86.3
Privacy from having others hear your consultation discussion	763	87.1	615	85.4	53	81.5	220	89.1	1651	86.5
Availability of medicines/methods at this facility	568	64.8	508	70.6	42	64.6	136	55.1	1254	65.7
The hours of services at this facility	664	75.8	585	81.3	46	70.8	197	79.8	1492	78.2
The number of days services are available to you	714	81.5	592	82.2	49	75.4	210	85.0	1565	82.0
The cleanliness of the facility	666	76.0	598	83.1	55	84.6	204	82.6	1523	79.8
How the staff treated you	664	75.8	590	81.9	59	90.8	221	89.5	1534	80.4
Cost for services or treatments	638	72.8	565	78.5	0	0.0	161	65.2	1364	74.0
Cronbach’s alpha									0.8156	

### Mean satisfaction score

**Fig. 2:**
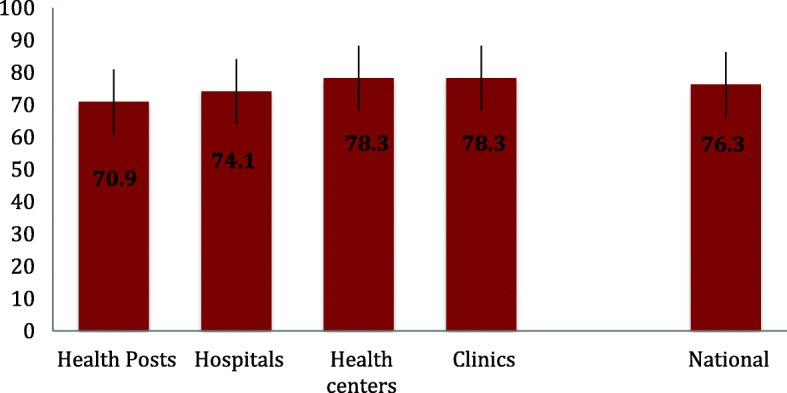
Satisfaction score (mean percent, 95% CI) on sick child care by facility type. Ethiopian Service Provision Assessment Plus Survey 2014

Figure 2 describes the mean satisfaction score by facility type. At national level the mean satisfaction score was 76%. Compared to mothers/caregivers who sought care at other types of facilities, those that sought care at health posts reported the lowest mean satisfaction score (71%).

### Assessments, examinations, and treatment of sick children, classified by diagnosis or major symptoms

Table 4 describes other process indicators for the management of sick children. Among children ultimately diagnosed with pneumonia, respiratory rate was counted in 56% of the cases and temperature was checked in 77%. Overall, 19% of children diagnosed with pneumonia were either hospitalized or referred elsewhere. Eighty-four percent were given some form of antibiotics (9% received injectable antibiotics, and 78% an oral antibiotic). Among the children diagnosed with bronchial spasm or asthma, 43% had their temperature checked, only 13% were checked for their respiratory rate, and 89% received antibiotics. Providers prescribed any antibiotics for 63% of children diagnosed with cough or other upper respiratory illness.

**Table 4:** Adherence to IMCI assessment, physical examination, and treatment among sick children. Ethiopian Service Provision Assessment Plus Survey 2014

Components of consultation	Pneumonia/ Broncho- pneumonia	Bronchial spasm/asthma	Cough or other upper respiratory illness	Fever	Measles	Malaria^d^	Any diarrhea without dehydration	Any diarrhea with dehydration	Ear infection	Malnutrition	All observed children
**IMCI assessment**											
3 main symptoms^a^	55	53	55	27	61	51	65	43	35	49	44
3 general danger signs^b^	3	0	6	3	0	4	1	23	8	6	4
Current eating or drinking habits	30	45	25	25	30	28	25	50	20	62	27
Caretaker advised to continue feeding and to increase fluid intake	12	3	16	19	22	23	20	43	17	38	17
**Physical examination**											
Temperature	77	43	87	92	67	91	88	74	72	74	74
Respiratory rate	56	13	48	47	46	32	22	11	16	31	29
Dehydration	20	9	17	38	60	28	56	54	19	29	23
Anemia	33	25	24	47	38	39	43	44	35	37	30
Ear (looked in ear/ felt behind ear)	9	37	13	11	9	11	14	0	69	7	11
Edema	4	9	3	10	9	7	0	13	3	35	7
Referred for any laboratory test	18	19	18	33	24	43	16	27	13	7	21
**Treatment**											
Referred outside or admitted	19	0	5	3	22	2	1	17	11	21	9
Any antibiotic	84	89	63	60	94	52	62	80	92	43	58
Injectable antibiotic	9	8	2	3	26	2	1	11	15	4	4
Oral antibiotic	78	80	62	59	90	50	61	76	86	41	56
Any antimalarial	3	0	3	6	3	36	0	0	2	2	4
ACT	1	0	1	1	3	16	0	0	0	1	2
Oral non-ACT	2	0	2	4	0	18	0	0	1	1	2
Injectable artesunate	0	0	0	0	0	0	0	0	0	0	0
Quinine	0	0	0	1	0	1	0	0	0	0	0
Oral bronchodilator	5	46	1	0	0	0	0	0	0	0	1
Oral medication for symptomatic treatment	47	46	48	65	42	55	23	26	40	12	35
Oral rehydration solution (ORS)	18	1	8	33	31	22	76	67	14	16	19
Intravenous fluid	0	0	0	0	0	0	0	0	0	0	0
Zinc	7	1	2	8	1	10	13	8	3	9	6
Described signs or symptoms requiring immediate return	21	16	21	24	0	30	20	17	16	16	19
Discussed follow-up visit	17	24	29	21	1	29	42	28	35	26	25
Number of children^c^	299	11	289	197	8	201	72	24	58	146	1908

Note: *ACT* artemisinin combination therapy

^a^ The three IMCI main symptoms are cough/difficulty breathing, diarrhea, and fever

^b^ The three IMCI general danger signs are inability to eat/drink anything, vomiting everything, and febrile convulsion

^c^ A child may be classified under more than one diagnosis; therefore, the numbers in the individual columns are not mutually exclusive and may add to more than the total number of observed children

^d^ Malaria reflects the provider-reported diagnosis, which may have been based on rapid diagnostic test (RDT), microscopy, or clinical diagnosis. The interviewing team did not verify this information

A majority of children (92%) diagnosed with fever had their temperature taken. Only 3% of children with fever were either referred or admitted, and 60% received antibiotics.

Among children diagnosed with malaria, 51% were assessed for all three IMCI main symptoms, and 4% were assessed for all three IMCI general danger signs. In addition, temperature was assessed in 91%, and anemia was assessed in 39% of the children. Overall, providers either hospitalized or referred the child elsewhere in 2% of these cases and gave some form of antimalarial medicine to 36%. The providers gave some antibiotics in half (52%) of these cases.

There were two categories of diarrhea: (1) any diarrhea without dehydration, and (2) any diarrhea with dehydration. The providers assessed dehydration in 56% of cases in the first category and in 54% of cases in the second category. Only 1% of children in the first category and 17% of children in the second category were admitted or referred to another facility. Oral Rehydration Solution (ORS) was prescribed for 76% of children in the first category (diarrhea without dehydration), while none received intravenous fluids. Among children in the second category (diarrhea with dehydration), 67% were given ORS while none were put on intravenous fluids. More children in the first category (diarrhea without dehydration) were given zinc (13%) than children in the second category (8%).

### Determinants of mothers or caretakers’ satisfaction with the quality of care provided to their sick children

Results from the multivariable regression models for the associations between structural and process quality and client satisfaction are presented in Table 5. A few results were consistent across the analyses. The number of basic amenities in the facility was negatively associated with clients’ satisfaction. Private facilities combined with providers’ sick child care training in the past 2 years were positively associated with client satisfaction. Structural and process composite indicators were not associated with client satisfaction with sick child health services.

**Table 5:** Multivariable analysis to identify factors associated with client’s satisfaction with sick child services. Ethiopian Service Provision Assessment Plus Survey 2014

Facility/provider characteristics	Model I	Model II			Model III		Model IV	
	β Coeff	95% CI	β Coeff	95% CI	β Coeff	95% CI	β Coeff	95% CI
Facility managing authority								
Public	Reference		Reference		Reference			
Private or Non-governmental organization	0.44 *	(0.26, 0.63)	0.43 *	(0.25, 0.60)	0.44 *	(0.26, 0.63)		
Urban/Rural								
Urban	Reference		Reference		Reference			
Rural	0.04	(−0.09, 0.17)	0.09	(−0.02, 0.21)	0.04	(− 0.09, 0.16)		
Facility type								
Hospitals	Reference		Reference		Reference			
Health centers	−0.08	(− 0.23, 0.06)	0.06	(− 0.06, 0.18)	− 0.09	(− 0.23, 0.06)		
Health posts	− 0.20	(− 0.53, 0.14)	0.08	(− 0.19, 036)	− 0.19	(− 0.53, 0.14)		
Clinics	−0.35 *	(− 0.62, − 0.08)	−0.20	(− 0.40, 0.002)	−0.35*	(− 0.62, − 0.08)		
Provider in the facility received sick child care training in the past two years								
No	Reference		Reference		Reference			
Yes	0.11 *	(0.02, 0.21)	0.11 *	(0.01, 0.20)	0.11 *	(0.02, 0.20)		
Caregivers education level								
No formal education	Reference		Reference		Reference			
Primary	0.01	(−0.10, 0.13)	0.01	(− 0.11, 0.12)	0.02	(−0.10, 0.13)		
Secondary	0.10	(−0.03, 0.23)	0.08	(−0.05, 0.20)	0.10	(−0.03, 0.22)		
Higher	0.002	(− 0.14, 0.15)	−0.01	(− 0.15, 0.13)	0.005	(− 0.14, 0.15)		
Structure composite indicator							0.03	(− 0.01, 0.08)
Routine management meetings	0.06	(−0.13, 0.26)			0.07	(−0.13, 0.26)		
System to collect client opinion	−0.08	(− 0.22, 0.06)			− 0.08	(− 0.22, 0.06)		
Quality assurance system	0.03	(−0.09, 0.14)			0.03	(−0.09, 0.14)		
Supervision	0.16	(−0.03, 0.35)			0.16	(−0.03, 0.35)		
Health workers always available	−0.10	(− 0.29, 0.10)			− 0.10	(− 0.29, 0.09)		
IMCI guide followed	−0.01	(− 0.13, 0.10)			− 0.02	(− 0.13, 0.10)		
Diagnose and/or treat malaria	0.20	(−0.21, 0.61)			0.21	(−0.20, 0.62)		
Number of basic amenities	−0.08*	(− 0.13, − 0.03)			− 0.08*	(−0.13, − 0.03)		
Number of infection prevention precautions	−0.02	(− 0.06, 0.02)			− 0.02	(− 0.06, 0.02)		
Number of sick child equipment available	0.03	(−0.02, 0.07)			0.03	(−0.02, 0.07)		
IMCI mother’s card	0.10	(−0.01, 0.20)			0.10	(−0.007, 0.20)		
Process composite indicator							0.04	(−0.003, 0.09)
Provider used visual aids			0.11	(−0.21, 0.43)	0.14	(−0.18, 0.46)		
Provider discussed follow-up visit			0.06	(−0.05, 0.17)	0.04	(−0.07, 0.15)		
Number of symptoms checked			0.01	(−0.02, 0.04)	0.01	(−0.02, 0.04)		
Number of physical examinations of sick child			0.004	(−0.02, 0.03)	0.002	(−0.02, 0.02)		
Number of information provided to caregiver			−0.02	(−0.05, 0.02)	− 0.01	(− 0.05, 0.02)		
**Random effect (region)**								
Variance (SE)	0.07 (0.04)	(0.03, 0.19)	0.08 (0.04)	(0.03, 0.21)	0.07 (0.04)	(0.03, 0.18)		
ICC	0.069		0 .079		0.068			
**Model fitness**								
AIC	5349.299		5353.751		5356.999		5416.084	
BIC	5482.59		5448.166		5518.06		5438.299	

*P-value< 0.05, **p-value< 0.001

*CI* Confidence Interval, *SE* Standard Error, *AIC* Akaike Information Criterion, *BIC* Bayesian information criterion, *LL* Log Likelihood

## Discussion

The assessment of sick children in Ethiopia was of low quality, especially in clinics and health posts, but most clients were satisfied with the service they received. Health posts and clinics scored higher in some of the satisfaction attributes (waiting time, health worker’s ability to discuss, and explanation on treatment provided) compared to hospitals and health centers. The average satisfaction score did not vary across facility types. Structural and process composite indicators were not associated with satisfaction of clients in sick child services.

This study is the first effort to assess the quality of care provided to sick children in Ethiopia combined with an analysis of the association of client satisfaction with a range of process and structural characteristics based on a nationally representative sample. The study included a large number of observations. The sampling design assumed that hospitals, health centers and clinics played a more important role than health posts in the national health system. Therefore hospitals, health centers, and private clinics were over-sampled to improve the precision of the survey in the assessment at these levels.

The study involved observations of client-provider interactions during data collection, making it susceptible to the so-called Hawthorn effect. This implies that behavior might have changed when being observed resulting in better performance than usual. The health workers, who were selected as observers, had prior experience of the IMCI strategy and guidelines. They were trained for 4 weeks, including practical demonstrations of assessments, physical examination, and treatment.

The three main symptoms in the IMCI assessments (difficulty breathing, diarrhea, and fever), and the three general danger sign (inability to eat or drink anything, vomiting everything, and febrile convulsion) [17] were frequently neglected in the assessment of sick children. Similar gaps in following the steps of the IMCI guidelines were observed in a multi-country study conducted in Namibia, Kenya, Tanzania and Uganda [18]. The methodology used in these studies was similar to our study. A child with general danger signs should be considered as a serious problem. Most children with a general danger sign need immediate referral or admission to hospital [17]. They may also need lifesaving treatments with injectable antibiotics, oxygen or other treatments, which may not be available in the facility.

All children with respiratory problems should have had their respiratory rate counted [19]. We found inadequate physical examination of cases with suspected pneumonia. In general, none of the upper respiratory illnesses should be treated with antibiotics [19]. In this study, an even higher amount of antibiotic use was seen in cases with cough or other upper respiratory symptoms. Similarly, inappropriate antibiotics prescription was observed in a study in Benin [20]. With the worldwide growing problems of antibiotic resistance, an appropriate use of antibiotics should be encouraged to ensure that these drugs are not overused.

A recent study revealed that facility infrastructure was poorly associated with quality of child health services [21], which indicates that availability of equipment and supplies does not guarantee good quality of care and satisfaction. However, it is difficult to provide good quality care without access to basic equipment [21, 22]. These findings are in line with our results, where the composite structural index was unrelated to client satisfaction. We also found that the number of amenities in a facility was negatively associated with satisfaction. In addition, a relatively low alpha value for the structural and process indicators might contribute to the poor association. A study conducted on facility assessment tools indicated that a quarter of quality measures were not assessed by any of the tools including SPA [23]. Our study did not show an association between the number of physical examinations performed on the sick child and client satisfaction. A study conducted in Paraguay showed a positive association between the number of examinations performed and patient satisfaction [24].

Patient satisfaction is an increasingly common component in estimating the quality of healthcare. A recently published study in Nigeria revealed that patient satisfaction measured at the facility immediately after a visit tends to be high, irrespective of the objective quality of the facility [25]. Another study in Nigeria also indicated that patient satisfaction must be complemented with additional objective measures [26]. This points at the need of better measures when moving forward to improve quality of care. With the new sustainable development goals and commitments for universal health coverage, it is essential that the quality of health care dimension should be added to the agenda.

Another limitation of our study was that outcomes were measured solely on client satisfaction and did not consider other health-related outcomes of the children. Client satisfaction is often biased by people’s own expectations of what constitutes good quality of care. Populations with low expectations are more likely to be satisfied with poor quality of care, undermining the demand side efforts to deliver high quality of care [27].

Our results have a number of policy implications. There was a poor association between structure and process composite indicator and client satisfaction. Other dimensions of quality of care that may influence client satisfaction should be included. The context within which healthcare is delivered is important for its quality [21]. There is also a need to improve health providers’ skills to carefully take the patient’s history and perform physical examination. This could be achieved through targeted training.

## Conclusions

Hospitals and health centers scored higher than health posts in most of the equipment and infrastructure indicators as well as adherence to clinical guidelines. However, health posts and clinics score higher in some of the satisfaction items. Quality gaps were observed in assessments, examinations, and treatment provided to sick children. The study also revealed that satisfaction of clients in sick child services in Ethiopia was not associated with composite indicators of equipment, infrastructure, and adherence to clinical guidelines. These findings highlight the need to include the context of the health system, in addition to structural factors and process indicators that may influence quality of sick child care and client satisfaction.

## Data Availability

The Ethiopian Public Health Institute (EPHI) primarily collected the data for this manuscript. Interested researchers may contact the focal person, Mr. Abebe Bekele, Director of Health Systems and Reproductive Health Research directorate at the EPHI, Addis Ababa, Ethiopia, through email abebe1277belay@gmail.com.

## Abbreviations

AIC: Akaike’s information criterion
BIC: Bayesian information criterion
HEW: Health Extension Worker
iCCM: Integrated Community Case Management
IMCI: Integrated Management of Childhood Illnesses
IMNCI: Integrated Management of Newborn and Childhood Illness
ORS: Oral Rehydration Solution
SARA: Service Availability and Readiness Assessment
SPA+: Service Provision Assessment Plus Survey
UNICEF: United Nations International Children’s Emergency Fund
WHO: World Health Organization
